# Multi-Objective Aerodynamic Optimization of the Streamlined Shape of High-Speed Trains Based on the Kriging Model

**DOI:** 10.1371/journal.pone.0170803

**Published:** 2017-01-27

**Authors:** Gang Xu, Xifeng Liang, Shuanbao Yao, Dawei Chen, Zhiwei Li

**Affiliations:** 1 Key Laboratory of Rail Traffic Safety of Ministry of Education; School of Traffic and Transportation Engineering, Central South University, Changsha, Hunan,China; 2 CSR Qingdao Sifang Co. Ltd., Qingdao, China; Beihang University, CHINA

## Abstract

Minimizing the aerodynamic drag and the lift of the train coach remains a key issue for high-speed trains. With the development of computing technology and computational fluid dynamics (CFD) in the engineering field, CFD has been successfully applied to the design process of high-speed trains. However, developing a new streamlined shape for high-speed trains with excellent aerodynamic performance requires huge computational costs. Furthermore, relationships between multiple design variables and the aerodynamic loads are seldom obtained. In the present study, the Kriging surrogate model is used to perform a multi-objective optimization of the streamlined shape of high-speed trains, where the drag and the lift of the train coach are the optimization objectives. To improve the prediction accuracy of the Kriging model, the cross-validation method is used to construct the optimal Kriging model. The optimization results show that the two objectives are efficiently optimized, indicating that the optimization strategy used in the present study can greatly improve the optimization efficiency and meet the engineering requirements.

## Introduction

The development of high-speed train technology indicates the level of high-tech development of a country. Currently, high-speed trains in China run very close to the ground or along the track at an actual operating speed of approximately 300 km/h, with a draw ratio that is much larger than the ratios of other means of transportation. At high-speed operation, the trains experience more complex aerodynamic characteristics [1–4]. The aerodynamic drag and lift greatly affect the economy and comfort of running trains. The research and development of high-speed trains has shown that streamlined head shapes are critical for the aerodynamic performance of trains. Streamlined design, especially the streamlined head shape design, of high-speed trains remains an important issue in high-speed train research. The optimum shapes can greatly improve the aerodynamic performance of high-speed trains. The aerodynamic drag, the lift, the lateral wind safety performance, the train crossing performance, the aerodynamic performance when passing through tunnels, the aerodynamic noise, and other factors [5–6] of running trains should be considered when designing high-speed train head shapes. Among these factors, the aerodynamic lift of the tail coach is the key aerodynamic load that affects the comfort and safety of running trains. Therefore, reducing the aerodynamic drag and lift of train coaches is the key issue for optimizing the streamlined head shape design of high-speed trains.

Experiment and numerical simulation are two methods currently used to study the aerodynamic performance of high-speed trains. The former includes full-scale train tests and wind tunnel tests of scale models [7–10]. The challenges of full-scale train tests include their long duration, large consumption of man power and material resources, and the restrictions of local conditions. The main challenge of wind tunnel tests is that the scale model must be compatible in geometric and flow conditions (including the Reynolds number and the boundary layer turbulence) with the real train. From the perspective of testing, the optimization of the streamlined train shape cannot reflect the nonlinear relationship between the key design parameters and the optimization objectives. With the development of computer technology, computational fluid dynamics (CFD) has been applied to the design, research and development of high-speed trains [11–13]. Compared with experiments, numerical simulation offers stronger controllability, allowing noise computation to be easily performed in short computing cycles. In addition, different conditions of incoming flow and aerodynamic characteristics under various parameters, especially those in difficult-to-operate working conditions, can be predicted. From the perspective of optimization, CFD offers incomparable advantages: by combining CFD technology with the mainstream optimization algorithm, we can achieve high-efficiency aerodynamic shape optimization design of high-speed trains.

Sun et al. [14] conducted optimization design for aerodynamic drag reduction of the nose shape and the upper wall height in the cab of CRH3 train. They used the optimization software model FRONTIER and the integrated software SCULPTOR and FLUENT, and the aerodynamic drag was the only optimization objective. The grid deformation technique of SCULPTOR helped to compute the grid deformation in the flow field, and FLUENT helped to calculate the aerodynamic drag values under each group of optimization design variables. As a result, the solutions of the optimization objective were obtained. Liu Jiali et al. [15] took the aerodynamic load and the aerodynamic noise source as the optimization design objectives; they used Catia for streamline modelling design, ICEM for automatic grid division, and FLUENT for aerodynamic characteristic analysis to complete the streamlined multi-objective optimization. Yu Mingge et al. [16] took the side force and the lift as their optimization design objectives and conducted automatic optimization design of the head shape of high-speed trains. Their optimization design process mainly established the three-dimensional parameterization model, aerodynamic grid division, aerodynamic numerical computation, vehicle dynamic computation, and the multi-objective aerodynamic optimization algorithm for high-speed trains. Li Ming et al. [17] established an automatic computing optimization analysis process for the aerodynamic performance of the head shape for a parameter-driven high-speed train. The overall multi-objective optimization design method based on the multi-objective elitist non-dominated sorting genetic algorithm II (NSGA-II) was used to perform the optimization design of the slenderness ratio of the train head, the longitudinal symmetric line, the maximum horizontal profile, the horizontal contour line of the coach bottom, the auxiliary profile line, the nose height of the train head, and other critical control variables related to aerodynamic performance. An aerodynamic head shape with better comprehensive performance was suggested. Overall, in the process of optimization design in the above studies, CFD analysis must be performed for each design point, which requires huge computing costs and greatly reduces the optimization efficiency.

As a result of the very complex shape of high-speed trains and the considerable amount of CFD computation required, surrogate models have been applied to aerodynamic shape optimization. Abroad, Krajnović [18] suggested using the response surface method to optimize the aerodynamic performance of trains, optimizing the crosswind stability and the aerodynamic drag separately. Three types of response surface models were studied, i.e., the polynomial function, the radial basis function neural network, and the combined model of the radial basis function neural network and the polynomial function. The existing studies show that combined models present better optimization results. Liao Yanping et al. [19] combined two single-objective optimization processes, in which the micro-pressure wave caused by trains passing through a tunnel was taken as the first optimization objective; they obtained the optimum section rate of the streamlined part of the head train. When the section rate remains constant, the Kriging model and the three-dimensional vehicle modelling function (VMF) parameterization method can be used to perform the single-objective optimization design to reduce the aerodynamic drag of the head train. Yo-Cheon Ku et al. [20] took reducing the micro-pressure wave caused by a train passing through a tunnel as their optimization design objective. They used the Broyden-Fletcher-Goldfarb (BFGS) algorithm and the response surface model to complete the unconstrained single-objective optimum design of the section rate of the streamlined head train at different nose cone lengths. Jongsoo Lee et al. [21] used the support vector machine (SVM) response surface model and the sequential quadratic programming method to collect 9 design variables and to determine 100 sampling points. Considering the bow and buttock lines, they completed the single-objective optimization design of the aerodynamic shape of trains to reduce the micro-pressure wave. V. V. Vytla et al. [22] used the Kriging model and the genetic algorithm-particle swarm optimization (GA-PSO) hybrid algorithm, taking the reduction of the micro-pressure wave as their optimization objective, to complete the single-objective optimization of the aerodynamic shape design. In China, to reduce the resistance of the head shape of the CRH380A high-speed train, Yao et al. [23] proposed a free-form deformation-based local function parametric method that used a genetic algorithm-based response surface model based on a smoothing factor general regression neural network to optimize the nose shape of the train. This optimization reduced the aerodynamic drag of the train by 8.7%. Cui et al. [24] took the aerodynamic drag as their optimization objective and used the response surface method to perform the optimization design of a high-speed train head at 500 km/h; they reduced the aerodynamic drag coefficient by approximately 20%.

To improve the optimization efficiency without affecting the optimization accuracy, the Kriging surrogate model was used in this study to complete the multi-objective optimization of the aerodynamic drag of the train and the lift of the train coach. In this study, the construction approach of the Kriging surrogate model was improved to reduce the flow field computing times and to improve the optimization efficiency. As a result, the traditional method of the solution maximization was replaced with the cross-validation method to search for more reasonable model parameters. The final optimization results show that this construction approach successfully produces a Kriging model that can meet the design requirements for the prediction accuracy using fewer sampling points. Thus, the optimization design efficiency is improved. The optimization process decreases the aerodynamic drag of the train and the lift of the train coach, indicating that the proposed optimization process is effective and can provide a new concept for optimizing the aerodynamic shape of high-speed trains.

## Parameterization Method

### Parameterization of the local shape function

**Fig 1:**
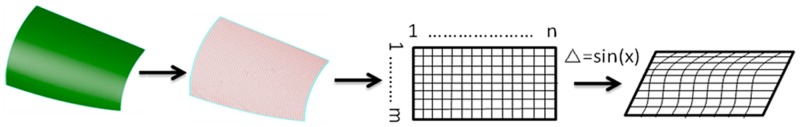
Curved surface deformation diagram of the local shape function.

Based on the basic idea of free deformation and the spline curve-surface method, the present study suggests the curved surface parameterization of the local shape function using key design points and the shape function to control complex curved surface deformation, according to the following steps:

1. For a given geometric shape, the area that needs local deformation is marked according to specific optimization requirements, so that smooth deformation of the geometric surface can be more easily achieved. When the overall deformation remains unaffected, the selected deformation area should ensure consistency of the boundary coordinate values, i.e., the coordinate values in each direction of the same boundary are equal.
2. Grid discretization is performed on the marked area to obtain the coordinate values of the discrete grid points in all areas. To achieve a smoother curved surface, the structural grid discrete method is used for grid discretization, as shown in Fig 1.
3. The deformation function is designed for each area and is selected at random, and smooth transitions should be ensured at the boundary of each area. For a regular boundary (where the coordinate values of discrete points in a certain direction remain unchanged), the coordinate values of the discrete points can be used as independent variables of the deformation function. For the irregular boundary (where the coordinate values of a discrete point in any direction are different), the topological numbers of the discrete points are the independent variables of the deformation function. Therefore, an irregular curved surface is projected onto a plane to form a regular rectangular area, as shown in Fig 1.
4. A weight factor *w*_*i*_ is set for each shape function. The algebraic sum of *w*_*i*_ determines the maximum deformation of the curved surface.
5. The incremental value of the coordinates Δ of all discrete grid points can be calculated using the shape function and the weight factor selected from each area.
6. When the incremental coordinate values Δ are algebraically added to the coordinate values of the original discrete grid points, the coordinate values of the deformed grid points can be obtained.
7. The deformed surface can be re-fitted according to the coordinate values of the grid points to complete the deformation.

Step (3) is key in the parameterization process. Different deformation functions will result in completely different deformations of the curved surfaces, and inappropriate functions can easily lead to pathological deformation. The frequently used deformation functions are the trigonometric function, exponential function, and logarithmic function; the polynomial function and spline function are more complex.

**Fig 2:**
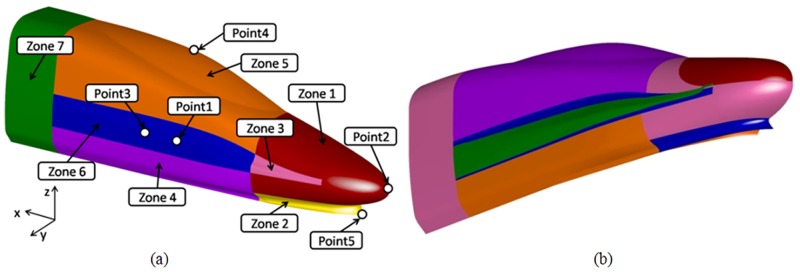
Deformation surfaces of the streamlined part. (a) deformation area of the streamlined part; (b) deformation in different areas.

Since the geometric shape is symmetric along the longitudinal section, the present study parameterizes one side of the longitudinal section of the streamlined part of the head train. After deformation, the longitudinal section becomes the symmetry plane, and the geometric shape of the other side is obtained. Thus, the parameterization design of the streamlined part of the head train is completed. The parameterization part is divided into seven deformation areas, as shown in Fig 2(a). Deformation areas 4, 5, 6 and 7 jointly control the train body width. The design parameter *w*_1_ is taken as the coordinate value of control Point 3 along the y direction. Deformation areas 5 and 6 control the perspective of the cab, and the design parameter *w*_2_ is taken as the coordinate value of control Point 4 along the z direction. Deformation areas 1 and 3 control the height of the nose cone, and the design parameter *w*_3_ is taken as the coordinate value of control Point 2 along the z direction. Deformation areas 3 and 6 control the drainage at the nose cone, and the design parameter *w*_4_ is taken as the coordinate value of control Point 1 along the y direction. For convenience in this study, when free deformation of the geometric surface within the design space remains unaffected, trigonometric functions are used as the deformation functions in all deformation areas. Fig 2(b) illustrates the deformations of the nose cone and the pilot, which show that the deformation areas are able to ensure fairness of the curved surface. In addition, smooth transitions between different deformation areas can be ensured.

### Overall optimization strategy

The Kriging model includes the regression model and the correlation model; the former presents a spatial global proximate, whereas the latter reflects the spatial distribution structure, which greatly influences the predictive ability of the Kriging model. The construction process of the Kriging model is an optimization process of the correlation model parameter *θ*_*i*_, i.e., the solution procedure of *θ*_*i*_ is translated into the non-restrictive and nonlinear maximized optimization through the likelihood estimation of the maximized response value. The idea of the cross-validation algorithm is used in the present study to minimize the prediction error of the training sample points when the optimum solution *θ*_*i*_ is obtained and the Kriging model is constructed. For the multi-objective optimization, selecting the relevant model parameters is a multi-variable, multi-objective optimization process. To simplify this problem, the multi-objective optimization process of the present study is translated into a single-objective optimization process. The target value with the largest variation range is chosen as the main objective for constructing the Kriging model. The prediction accuracy of other objective functions can be increased by increasing the prediction accuracy of the main objective. For the single-objective optimization process, the optimization parameter is obtained using the real number encoding-based genetic algorithm. Information of all given sampling points should be fully used to reduce the number of training sample points. In this study, the approach for constructing the Kriging model is designed based on the cross-validation algorithm, shown as follows:

1. The main objective is determined according to the variation range of the objective function value;
2. The value range of the relevant model parameter *θ*_*i*_ can be determined in accordance with the influences of the design parameter values on the objective function values;
3. All the initial parameter values required by the genetic algorithm are given, including the population size, the selective probability, the crossover probability, the mutation probability, the initial population, and the maximum evolution algebra;
4. The training sampling points are randomly divided into *N* groups. To improve the availability of the information about the sampling points, the number of sampling points in one group is limited;
5. Sampling points from *N −* 1 groups are chosen to construct a sub-Kriging model, for which the relevant parameter is the *θ*_*i*_ value of the population individual. The sampling points of the remaining group are used as sample checkpoints. When the *θ*_*i*_ value remains unchanged, each group of sampling points is used as a sample checkpoint in sequence.
6. The average value of the summation of the absolute values of the prediction error *error*_*i*_ of *N* groups of sample checkpoints is used as the objective function
7. The genetic algorithm is used for the optimization. Thus, the value of *θ*_*i*_ when $\sum_{i=1}^{N}\left|error_{i}\right|/N$ is the smallest is obtained. The model with the smallest prediction error among the *N* sub-Kriging models based on the *N* groups of sampling points is taken as the final model.

The above procedure shows that the amount of computation required for the construction approach based on the cross-validation algorithm is much greater than that required for the traditional construction approach. However, this difference is negligible when compared to a flow field computation. The advantage of this construction approach is presented in the aerodynamic shape optimization design.

**Fig 3:**
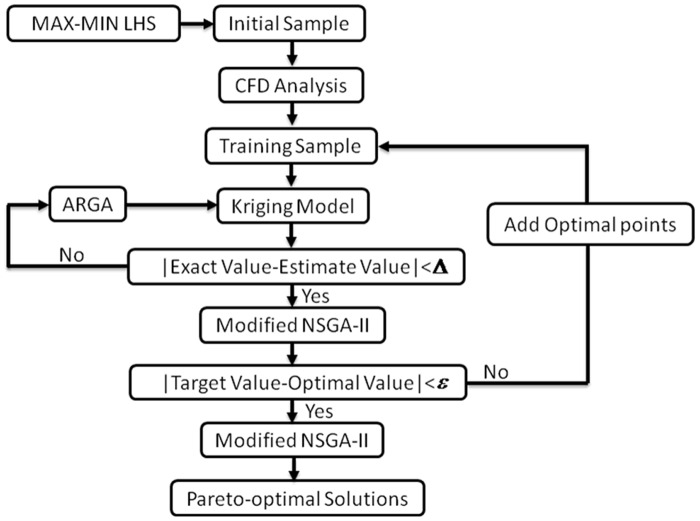
Multi-objective optimization design process.

The overall optimization process is shown in Fig 3. First, the Latin hypercube sampling method is used for sampling within the design space. Next, CFD flow field computation is used to obtain the accurate objective function value of the initial sampling points. A certain number of initial sampling points is chosen, and the genetic algorithm is used to train the Kriging model based on the cross-validation method. The Kriging model is trained to be the optimal one using the sampling points. Then, optimization is performed on the optimal model to determine the optimal point. When the optimum solution and the CFD results satisfy the error requirement, the optimal Kriging model is then completed. When the error requirement cannot be met, the optimization design points are expanded to include the initial sampling points, a new Kriging model is constructed, and a new round of training starts. The Kriging model and the multi-objective genetic algorithm are used for optimization within the design space. As a result, the Pareto-optimal solutions within the design space can be obtained, and the optimization process comes to an end. The multi-objective optimization for the drag of the whole train and the lift of the train coach is performed in this study and the optimization results were also analysed in detail.

## Geometrical Models and Numerical Details

**Fig 4:**
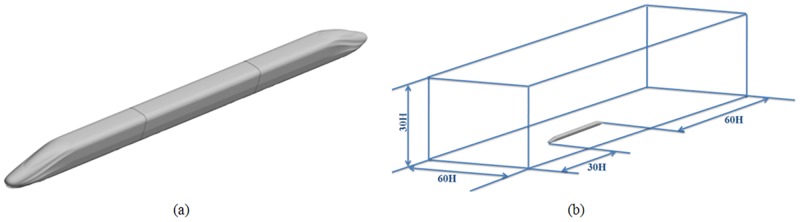
The model and the computational domain of EMU1. (a) EMU1 model of the whole vehicle; (b) Computational domain.

The three-coach marshalling model of the head coach, the middle coach, and the train coach is used to evaluate the head shape aerodynamic performance. The computational model is named EMU1. The model and the computational domain of the three-coach marshalling model EMU1 are shown in Fig 4(a).

To reduce the computation and time required for geometric modelling and computation, some locations are simplified, and the pantograph and other devices are deleted. Taking the train height H as the characteristic length, the distance between the entrance of the computational domain and the nose cone of the head train is approximately 30H, and the distance between the nose cone of the train coach and the exit of the computational domain is approximately 60H. The distance from the train centre to the boundary on both sides of the computational domain is approximately 30H; the distance from the ground to the top boundary of the computational domain is approximately 30H, as shown in Fig 4(b).

**Fig 5:**
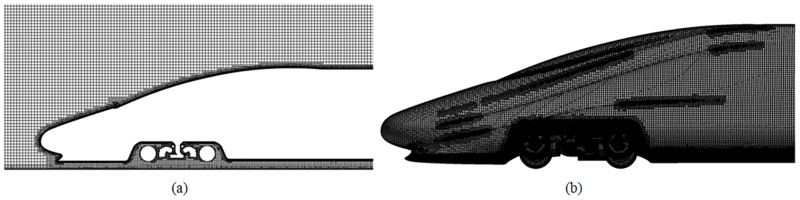
Grid diagram of the train head. (a) Longitudinal section of the train head; (b) Surface of the train head.

Grid division and grid quality play important roles in the computational efficiency and the astringency and precision of the computational results. Larger grids can be used in the whole computational domain, and the grid is refined in the areas where the flow field experiences large changes, including the areas around the train body and the wake flow. Fine grids can be transitioned to coarse grids in a layer-by-layer manner. The thickness of the first layer of the boundary layer next to the train is selected according to the principle that the computed Y+ value is within the range of 30–100. A reasonable number of boundary layers ensures that the size of the boundary layer gradually transitions to the size of the main grid. Cartesian grids are used to produce the boundary layer grids on the train surface and the ground, with a total thickness of 30 mm. To better connect to the hexahedral grids and to ensure a high grid quality, six boundary layers with an increment ratio of 1.2 are used, and the thickness of the first layer is 3.02 mm. The overall number of grids for the three-coach marshalling train is approximately 35 million. The grid diagram of the train head and the longitudinal section are shown in Fig 5.

The grids in this study account for the small features in positions such as the nose cone and the bogie. When the aerodynamic force is analysed, we use relative motion to simulate the external flow field near the train. The train is set to be static, and the velocity inlet boundary is set with the incoming air flow in the direction opposite to the train running direction but at the same speed, i.e., 300 km/h; the exit uses the pressure outlet boundary. The train surface uses the fixed wall boundary conditions, while the ground uses the moving wall boundary conditions.

Since the Mach number corresponding to the train speed is approximately 0.25, the airflow can be taken as an incompressible fluid for the solution. The incompressible steady Reynolds average algorithm is used for computation in the present study. The SIMPLE algorithm is used for the pressure-velocity coupling, and the shear stress transport (SST) k-ω model is used as the turbulent model. The Wilcox k-ω model is used close to the wall, while k-ε models are used for the boundary layer and for the free shear layer, transitioning with a blending function; the two-equation eddy viscosity model of the incompressible/compressible turbulence is used between the integration and the wall surface. For the (Navier-Stokes) NS discrete equation, the second-order upwind scheme is used to discretize the convective term, and the second-order central differencing scheme is used to discretize the dissipative term.

## Results Analysis

When optimization is performed based on the Kriging response surface, the parameters for the genetic algorithm are set to an initial population of 200 and a maximum evolution algebra of 1000. The selection operator applies the roulette wheel method, with a crossover probability of 0.9 and a mutation probability of 0.3.

**Fig 6:**
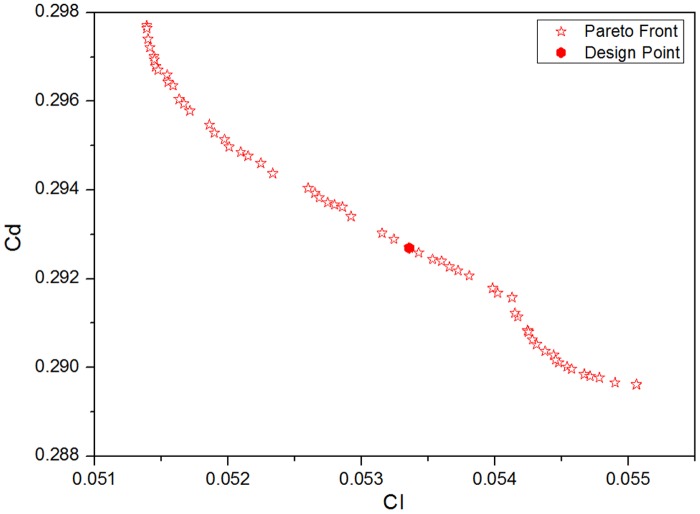
Pareto solutions based on drag of the whole train and the lift of the train coach.

After 1000 iterations, the Pareto-optimal solutions are quite stable and are distributed as follows:

Fig 6 shows that the drag coefficient of the whole train varies between 0.288 and 0.298, with a difference of approximately 3.3% between the maximum value and the minimum value. In addition, the lift coefficient of the train coaches varies between 0.051 and 0.054, with a difference of approximately 5.6% between the maximum value and the minimum value. Thus, these two values are quite sensitive to changes in the aerodynamic head shape. Because this study mainly focuses on the optimization of the aerodynamic drag of the train, the lift of the train coach is considered acceptable if the amplitude is no greater than that for the original shape. Using CFD analysis, the lift coefficient of the train coach for the original shape is approximately 0.064; in the Pareto-optimal solutions, all the lift coefficients of the train coach are smaller than this value. Therefore, the point in the centre of the above figure is chosen as the typical design point to verify the prediction accuracy of the Kriging model.

The numerical simulation results of the typical design points and the prediction results of the Kriging model are listed in the following table. Table 1 shows that compared to the original shape, the aerodynamic drag of the typical design decreases by 7.2% and that the lift of the train coach is much smaller than that of the original shape, with a maximum error of 0.445%. The predicted results and the computational results for the lift of the train coach vary somewhat but show a maximum error of only 1.36%, satisfying the engineering requirements. Therefore, the Kriging surrogate model is considered to be able to reflect the relation between the design parameters and the optimization objectives.

**Table 1:** Numerical simulation results of typical design points and prediction values of the Kriging model.

	Real Cl	Kriging	Error	Real Cd	Kriging	Error
Test Case	0.0541	0.05336	1.36%	0.2914	0.2927	0.445%

The variation values of the parameters of typical design points relative to the original shape are shown in Table 2. The width of train body of the streamlined part is controlled by *w*_1_; the perspective of the cab is controlled by *w*_2_; the height of the nose cone is controlled by *w*_3_; and the outline of the diversion trench is controlled by *w*_4_. Therefore, Table 2 shows that the width of the streamlined part of the optimal shape and the width of the diversion trench decrease, while the height of the nose cone and the perspective of the cab increase slightly.

**Table 2:** Variation values of the parameters of the typical design points relative to the original shape (1:1).

	*w*_1_/mm	*w*_2_/mm	*w*_3_/mm	*w*_4_/mm
Test Case	-25.6371	11.9675	98.43613	-69.5041

**Fig 7:**
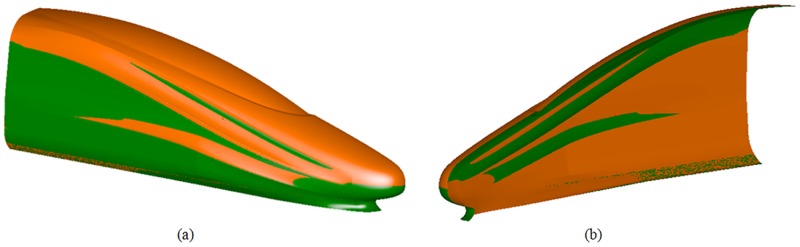
The original shape vs. the optimal shape. (a) the outside-in perspective; (b) the inside-out perspective.

In Fig 7, the green area is the original shape, and the orange area is the optimal shape. This figure shows that after optimization, the geometric shape of the train stays approximately the same, the bottom width of the streamlined part and the pilot remain approximately the same, the cab and the nose cone are lifted up, the width of the streamlined part narrows, and the diversion trench narrows slightly.

The aerodynamic force of the original shape and the typical design points are listed and compared in Table 3. Optimization improves the aerodynamic performance of the typical design points to different degrees, decreases the lift of the train coach by 15.9%, and decreases the aerodynamic drag of the train by 7.2%. For the original shape, the shear drag and the pressure drag are quite similar, with the latter being slightly larger. The pressure drag of the typical design points decreases greatly, and the shear drag increases slightly. Optimizing the drag reduction of the aerodynamic shape mainly comes from reducing the pressure drag of the train.

**Table 3:** Aerodynamic force of the original shape and the typical design points.

	Tail Cl	Total Cd	Pressure drag	Shear drag
Original	0.064	0.314	0.175	0.139
Test Case	0.0541	0.2914	0.1499	0.1415
Reduction	15.9%	7.2%	9.15%	-1.8%

**Fig 8:**
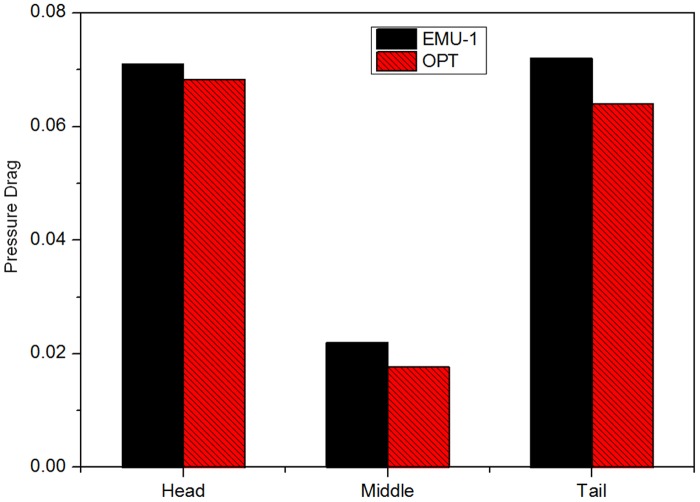
Comparison of the pressure drag coefficients of each coach before and after optimization.

To better understand the aerodynamic performance of the train after optimization and to determine the influence of changing the aerodynamic shape of the streamlined part on the other parts of the train body, the pressure drag coefficients of each coach before and after optimization are shown in Fig 8, and pressure drag mainly exists on the nose cone and the tail cone.

After optimization, the pressure drag of the three coaches decreases greatly. The pressure drag of the nose cone decreases by 9.3%; the pressure drag of the tail cone decreases by 11.11%; and the overall pressure drag decreases by 9.15%. Optimizing the aerodynamic drag mainly comes from reducing the pressure drag.

**Fig 9:**
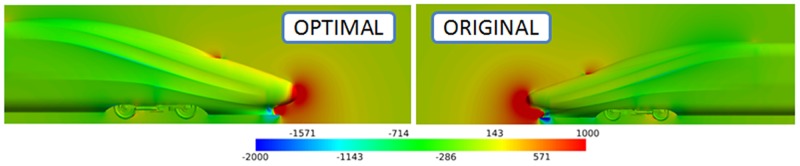
Pressure distribution near the head coach for the original shape and the optimal shape.

The drag difference between the optimal shape and the original shape is analysed from the perspective of the pressure distribution. The pressure distribution near the head coach is shown in Fig 9, and a large area of high pressure exists near the nose and the pilot.

There is an intermediate pressure area in the transition region between the nose cone and the glass of the cab, and an obvious low pressure area exists at the bottom of the pilot. After optimization, the height of the nose cone increases slightly, while the window of the cab is lifted such that it decreases the magnitude of the cab inclination. In addition, the high pressure area in front of the nose cone is smaller than that of the original shape. A wide range of high pressure area exists between the nose cone and the ground of the original shape, resulting in more pressure drag for the original shape than for the optimal shape.

**Fig 10:**
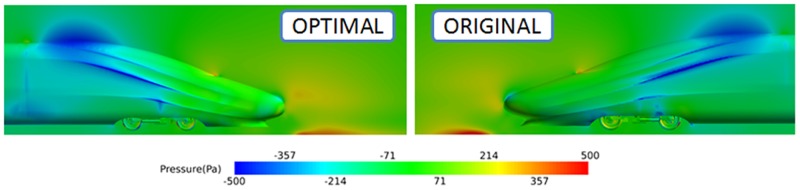
Pressure distribution near the tail coach of the original shape and the optimal shape.

Similarly, the pressure distribution near the nose cones of the train coaches of the optimal shape and the original shape are compared in Fig 10.

The pressure distribution of the tail indicates that a high pressure area with a slightly higher amplitude exists on top of the nose cone of the optimal shape. The positive pressure gives a forward push to the nose cone of the train coach, lowering the pressure drag.

**Fig 11:**
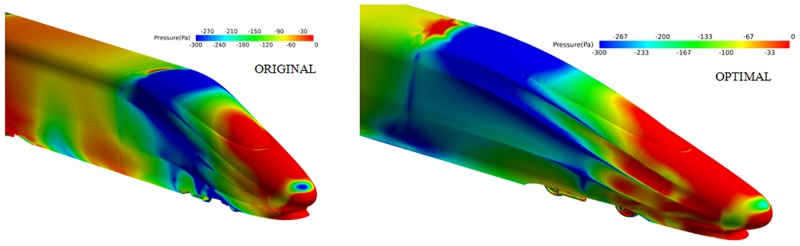
Pressure distribution on the surface of the train coaches of the original shape and the optimal shape.

To better study the difference between the lifts of the train coaches of both the original shape and the optimal shape, the pressure distribution on the surface of the nose cone of the train coaches of the two shapes is shown in Fig 11.

Optimization greatly increases the positive pressure over the diversion trench. The positive pressure directly above the surface of the nose cone pushes the nose cone down, effectively reducing the lift of the nose cone of the train coach.

**Fig 12:**
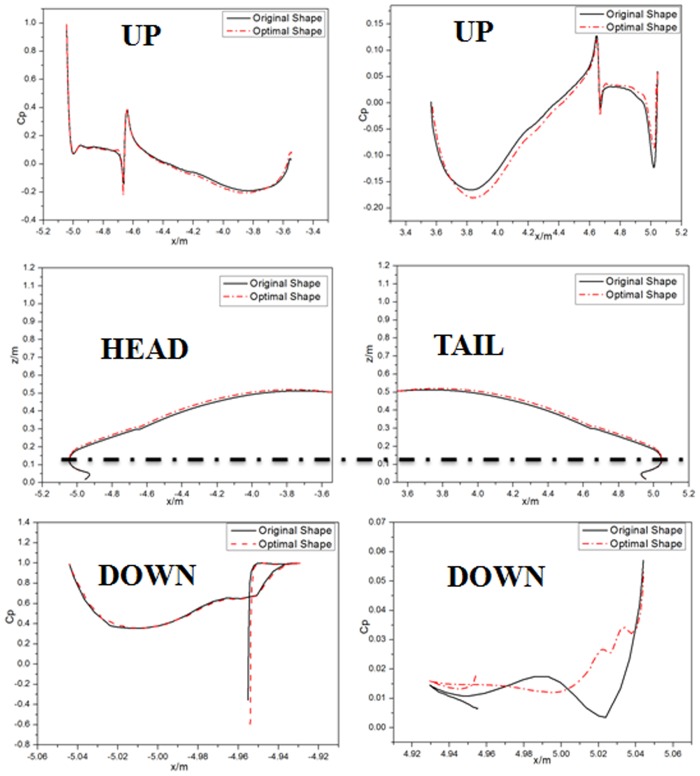
Comparison of the drag coefficients on the streamlined longitudinal section of the head and the tail before and after optimization.

To explicitly study the differences resulting from changing the streamlined shape of the optimal shape and the original shape, the drag coefficients on the streamlined longitudinal section of the head and the tail before and after optimization are shown in Fig 12.

The pressure drag difference mainly exists on the train coach. The negative surface pressure on the train coach with the optimal shape increases slightly in the form of train operating drag. Since the increasing amplitude of the negative drag is limited and because there is a large inclination angle on the upper surface, the drag increase is limited. Comparatively speaking, the drag corresponding to the lower surface in the form of a large pushing force is clearly larger than that of the original shape. In addition, the lower surface is vertical to the flow direction, resulting in a smaller pressure drag of the train coach, and the total pressure drag is therefore optimized.

## Conclusions

To reduce the computational times of the flow field and to improve the optimization efficiency, the construction method of the Kriging surrogate model is improved in this study. The traditional maximization of the solution is replaced with the cross-validation method to search for more reasonable model parameters. The final optimization results show that this construction method uses fewer sampling points to complete a Kriging model with a prediction accuracy that satisfies the design requirements. Thus, the optimization design efficiency is improved. A Pareto solution set related to the aerodynamic drag and lift of the train are found in the design space based on the new Kriging model and the multi-objective genetic algorithm. A typical design point is chosen for numerical simulation and compared with the aerodynamic performance of the original EMU1 shape. The drag of the typical design point is reduced by approximately 7.2% compared to the original shape. The lift of the train coach is 15.9% smaller than that of the original shape, indicating that the optimization process is efficient enough to be used for the future aerodynamic shape optimization of high-speed trains.
